# Fgf2 is expressed in human and murine embryonic choroid plexus and affects choroid plexus epithelial cell behaviour

**DOI:** 10.1186/1743-8454-5-20

**Published:** 2008-12-29

**Authors:** Sarah Greenwood, Adam Swetloff, Angela M Wade, Tetsuya Terasaki, Patrizia Ferretti

**Affiliations:** 1 DevelopmentalBiology Unit UCL Institute of Child Health, 30 Guilford Street, London WC1N 1EH, UK; 2 Paediatric Epidemiology & Biostatistics, UCL Institute of Child Health, 30 Guilford Street, London WC1N 1EH, UK; 3 Membrane Transport and Drug Targeting Laboratory, Graduate School of Pharmaceutical Sciences, Tohoku University, Aoba, Aramaki, Aoba-ku, Sendai 980-8578, Japan; 4 Department of Applied Biological Sciences, School of Community and Health Sciences, City University, 20 Bartholomew Close, London EC1A 7QN, UK; 5 Department of Genetic Medicine and Development, University of Geneva, 1 Rue Michel-Servet 1211 Geneva-4, Switzerland

## Abstract

**Background:**

Although fibroblast growth factor (Fgf) signalling plays crucial roles in several developing and mature tissues, little information is currently available on expression of Fgf2 during early choroid plexus development and whether Fgf2 directly affects the behaviour of the choroid plexus epithelium (CPe). The purpose of this study was to investigate expression of Fgf2 in rodent and human developing CPe and possible function of Fgf2, using *in vitro *models. The application of Fgf2 to brain *in vivo *can affect the whole tissue, making it difficult to assess specific responses of the CPe.

**Methods:**

Expression of Fgf2 was studied by immunohistochemistry in rodent and human embryonic choroid plexus. Effects of Fgf2 on growth, secretion, aggregation and gene expression was investigated using rodent CPe vesicles, a three-dimensional polarized culture model that closely mimics CPe properties *in vivo*, and rodent CPe monolayer cultures.

**Results:**

Fgf2 was present early in development of the choroid plexus both in mouse and human, suggesting the importance of this ligand in Fgf signalling in the developing choroid plexus. Parallel analysis of Fgf2 expression and cell proliferation during CP development suggests that Fgf2 is not involved in CPe proliferation *in vivo*. Consistent with this observation is the failure of Fgf2 to increase proliferation in the tri-dimensional vesicle culture model. The CPe however, can respond to Fgf2 treatment, as the diameter of CPe vesicles is significantly increased by treatment with this growth factor. We show that this is due to an increase in cell aggregation during vesicle formation rather than increased secretion into the vesicle lumen. Finally, Fgf2 regulates expression of the CPe-associated transcription factors, *Foxj1 *and *E2f5*, whereas transthyretin, a marker of secretory activity, is not affected by Fgf2 treatment.

**Conclusion:**

Fgf2 expression early in the development of both human and rodent choroid plexus, and its ability to modulate behaviour and gene expression in CPe, supports the view that Fgf signalling plays a role in the maintenance of integrity and function of this specialized epithelium, and that this role is conserved between rodents and humans.

## Background

The choroid plexus epithelium (CPe) is a specialized neuroepithelium that is involved in secretion of cerebrospinal fluid (CSF) into the cerebral ventricles and in maintaining the homeostasis of the brain during development and throughout life [1-3]. Most CSF is secreted by the CPe and is re-absorbed mainly at the site of the arachnoid villi. Knockout of genes expressed in the CPe, such as *E2f5*, a member of a family (*E2f1–E2f6*) of transcriptional regulators, *FoxJ1*, a member of the forkhead-box (Fox)/winged helix gene family, and *p73*, have been associated with hydrocephalus [4-6]. The mechanisms leading to the apparently non-obstructive hydrocephalus in these mutants appear to differ and have yet to be fully elucidated. These genes are expressed early during CPe development in rodent and humans [6-11] and *Foxj1 *has been reported to be down-regulated by Fgf2 (fibroblast growth factor 2) in neural cells [12].

Some biogenic amines, neuropeptides and hormones also have the ability to modulate the function of the CPe, including its secretory activity [13-17], but the effect of growth factors on its cellular function has not been well studied, especially in the embryonic CPe. Fgf2 has been implicated in the regulation of cell survival and apoptosis, adhesion, motility, and differentiation [18], and in the brain, among other effects, in the control of neural stem cell proliferation both during development and in the adult following intraventricular administration. It has also been suggested that Fgf treatment can stimulate neurogenesis and aid repair following brain injury [19-21]. Injection of Fgf2 into the ventricles of the brain, however, has been reported to induce hydrocephalus [22-24]. This could be a hindrance in the development of Fgf-based treatments [25], as the mechanisms underlying the effect of Fgf2 on CSF accumulation remain unclear. Given that Fgf can affect several cell types in the brain, induction of hydrocephalus following Fgf administration might not be due to a direct effect of Fgf on the CPe. On the other hand, Fgf2 might directly affect CSF secretion or reabsorption [23] or even CSF circulation. For example, defects in ciliogenesis, as reported in the *FoxJ1 *knock-out mouse, can affect CSF dynamics in the ventricles and result in hydrocephalus [5].

As members of the fibroblast growth factor receptor family (FgfR 1–4) are expressed in the CPe and are differentially regulated during development [26], it is likely that Fgf signalling can directly affect at least some aspects of CPe cell behaviour. While *in vitro *models cannot reproduce the complexity of the responses leading to the hydrocephalic phenotype *in vivo*, they can allow the direct effects of Fgf on CPe cells to be tested.

At least one FgfR ligand, Fgf2, is known to be expressed in the adult and foetal CPe [27-29], but its expression during early development has not been investigated either in rodents or humans. We have therefore assessed Fgf2 pattern of expression during mouse and human CP development in relation to proliferation. Having found that Fgf2 protein is expressed both in human and mouse embryonic choroid plexus, we have used embryonic CP cells cultured in Matrigel to investigate putative roles of Fgf2 on CPe behaviour. In this tri-dimensional matrix, embryonic CP cells form fluid-filled spherical structures called vesicles, and we have recently shown that their growth depends on secretion within the lumen [30,31]. The CPe phenotype and the behaviour of the cells in this culture system appear to be very similar to that *in vivo*, representing a valuable model to assess the effect of growth factors on cell-cell adhesion and cell migration as well as fluid secretion and solute transport [30]. This is further supported by this study, as we show that Fgf2 does not affect CPe proliferation *in vitro *in a tri-dimensional environment, consistent with the significant Fgf2 expression and limited proliferation observed in the choroid plexus *in vivo*. We also show that whereas Fgf2 treatment *in vitro *affects CPe vesicle formation in a dose-dependent fashion, it does not alter its secretory activity. Fgf2, however, can regulate expression of the CPe-associated transcription factors *E2f5 *and *FoxJ1*. Altogether this study suggests that Fgf signalling plays a role in CPe development and possibly in its integrity and homeostasis.

## Methods

### Tissue collection and reagents

Choroid plexus for cultures, and brains to be fixed in 4% paraformaldehyde and paraffin embedded for immunohistochemistry were dissected from out-bred CD-1 albino mice supplied by Charles River Mouse Farms (Kent, UK) and housed under Home Office regulations. The morning of detection of a vaginal plug was designated as embryonic day 0.5 (E0.5). Mice were killed by cervical dislocation according to Home Office regulations at the times indicated in the results section and embryos removed for immunohistochemical analysis or CPe culture preparation as described below. Paraffin sections from human embryos at 8 weeks of gestation obtained under ethical approval were provided by the Wellcome/MRC-funded Human Developmental Biology Resource. Unless otherwise specified, reagents used were from Sigma-Aldrich, UK.

### Choroid plexus cell cultures

The fourth ventricle CP from E12.5 mice was dissected and CP cells isolated and cultured as previously described [30,31]. Cells were grown in HEPES-buffered Dulbecco's modified essential medium (H-DMEM, Gibco-BRL, Paisley, UK) supplemented with 10% foetal calf serum (FCS) and penicillin-streptomycin (Gibco-BRL) either as monolayers on laminin (Sigma, UK) or seeded in Matrigel (Becton Dickenson, distributed by Stratech Scientific Ltd, Dudley, UK) to allow vesicle formation. In vesicle culture experiments 1–2 × 10^5 ^CPe cells were plated on to Matrigel in cloning cylinders of 8 mm diameter and fed every 2–3 days with the culture medium described above.

The TRCSFB-2 cell line was established from CPe cells isolated from a transgenic rat harboring the temperature-sensitive simian virus 40 (ts SV 40) large T-antigen gene [32]. Cells were grown in DMEM (Gibco-BRL) supplemented with 10% FCS and penicillin-streptomycin on collagen I (Vitrogen, Cohesion Technologies, Inc. CA, USA) at 33°C [32]. Before carrying out RNA expression studies, TRCSFB-2 cells were cultured for 24 h at 37°C.

### Cell analysis and treatments

CPe behaviour was monitored by regular observation under an inverted microscope (IM45 Zeiss) and by time-lapse photography for up to 4 consecutive days using Openlab software (Improvision, UK). For Fgf2 treatment, Fgf2 (R&D Systems, Oxford, UK) at the desired concentration was added to the culture medium either at the time of plating CPe cells or later as specified in the Results. To study the effect of Fgf2 on vesicle recovery after inhibition of secretion and collapse, cultures were treated for 48 h either with a combination of 100 μM acetazolamide and 5 mM ouabain or with the vehicle alone (DMSO controls).

### Proliferation analysis and immunohistochemistry

Fgf2 was detected by an anti-Fgf2 mouse monoclonal antibody (Transduction Laboratories, Lexington, UK, 1:100 dilution). Cell proliferation was detected by: anti-bromo-deoxyuridine (BrdU) rat monoclonal antibody to detect cells in S phase that had incorporated BrdU (Oxford Biotechnology, Oxford, UK, 1:100); anti-phosphorylated-histone 3 (p-H3) rabbit antibody to detect cells in M phase (Upstate Biotechnology, Buckingham, UK; 1:100 or 1:500 dilution, see below); a mouse monoclonal antibody to PCNA, an auxiliary factor for DNA polymerase delta, to detect cells in G1-S phase (clone PC10, Santa Cruz Biotechnology, Inc., CA, USA, 1:100). The secondary antibodies used, fluorescein-conjugated goat anti-rabbit (1:50 dilution), and peroxidase-conjugated goat anti-rabbit, goat anti-mouse and goat anti-rat immunoglobulins (1:100 dilution), were all from DAKO (Bucks, UK). Hoechst dye H33258 (1:500 dilution of a 1.2 mg/ml stock) was used to counter-stain nuclei.

Mitotic cells in CPe vesicles and aggregates were detected using the anti-p-H3 antibody. Cells grown in Matrigel inside cloning cylinders placed onto glass coverslips were fixed in 4% paraformaldehyde (PFA) for 10 min. Non-specific binding sites were blocked with 1% goat serum in PBS for 40 min. CPe vesicles/aggregates were incubated overnight at 4°C with the anti-p-H3 antibody, thoroughly washed and incubated with fluorescein-conjugated goat anti-rabbit for 30 min. Nuclei were counterstained for 15 min with Hoechst dye.

The anti-PCNA monoclonal antibody was used to stain primary CPe cells fixed for 10 min in 4% PFA. Bound antibody was detected using a fluorescein-conjugated goat anti-mouse antibody and nuclei counterstained with Hoechst dye. Paraffin sections were stained either with the anti-Fgf2 mouse monoclonal antibody or with the anti-p-H3 rabbit antibody (1:500 dilution) essentially as previously described [7,26].

Tissues and cells were viewed under a Zeiss Axioplan microscope and digitally scanned using a Hamamatsu digital camera (C4742-95, Hamamatsu Photonics KK, Japan) into Openlab software (Improvision Ltd, Coventry, UK).

### RT-PCR

TRCSFB-2 cells were trypsinised and the pellet stored in 1 ml TRI-Reagent at -20°C until RNA extraction following the manufacturer's protocol. cDNA was synthesised from 1 μg of RNA using M-MLV reverse transcriptase and random hexamers according to the manufacturer's instructions (Promega, Southampton, UK). The PCR step was performed using Taq polymerase at an annealing temperature of 57°C with specific primer pairs for each gene, which were designed to span intron-exon boundaries to exclude genomic DNA contaminations. The linearity range of amplification for each set of primers was evaluated and the number of cycles selected accordingly. The primers (Genosys, Cambridge, UK) used and number of cycles and product size (in brackets) are: *Gapdh *(20, 491 bp) *5' TTCCAGTATGACTCCACTCACG 3'*, *5' GGATGCAGGGATGATGTTCT 3'*; *E2f5 *(25, 227 bp) *5' TGTGGCTACAGCAAAGCATC 3'*, *5' GGCCCTGAGTGACTCTTCAG 3'*; *FoxJ1 *(25, 205 bp) *5' TACTGCTGACCCAGGAGGAG 3'*, *5' GGTAGCAGGGCAGTTGATGT 3'*, *TTR *(30, 505 bp) *5' CAGATCCACAAGCTCCTGAC 3'*, *5' CTGCTTTGGCAAGATCCTGC 3'*; p73 (30, 253 bp) *5' AGAGTGTGGTTGTGCCGTATG 3'*, *5' TCCCGGTAATGGTCTTCATC 3'*. PCR products were visualized on a 1.5% agarose gel containing ethidium bromide under ultraviolet light and imaged by using a Gel imaging system and Alphaease 3.3 software (GRI Ltd, Braintree, UK).

### Measurements and statistical analysis

The diameter of each vesicle was measured using an eyepiece graticule at ×20 magnification. Measurements were taken to within the nearest quarter graduation on the eyepiece. Each vesicle could be identified easily by recording the co-ordinates on a finely graduated x-y stage and was measured at 2-day intervals at approximately the same time of day on days 4, 6, 8, and 10. The diameters were recorded 'blind' to treatment group.

The number and size of aggregates and vesicles was measured using the Openlab 3.1.5 software. The size of aggregate and vesicles was estimated by measuring the surface area in μm^2 ^of their focal equatorial plane. The measurements were automatically recorded by the Openlab 3.1.5 programme, stored in Microsoft Excel and later transferred to SPSS Statistics 11.5 (SPSS Inc.) for statistical analysis. Aggregates and vesicles were measured in the same fields at different days. Four fields of view were taken on each measurement day. Cell motility was assessed following time-lapse photography by measuring the distance travelled by a cell using the Openlab 3.1.5 software.

Data are expressed as means ± standard error of the mean (mean ± SEM). Parametric and non-parametric tests as appropriate were used to compare treatment groups at different time points. Regression analysis was used to quantify the effects of treatments over time when different vesicles were assessed on each day. Multilevel modelling was used to model the serial changes in individual vesicles over time [33]. Results are presented with 95% confidence intervals.

## Results

### Expression of Fgf2 and the mitotic marker, p-H3 in developing CPe

**Figure 1:**
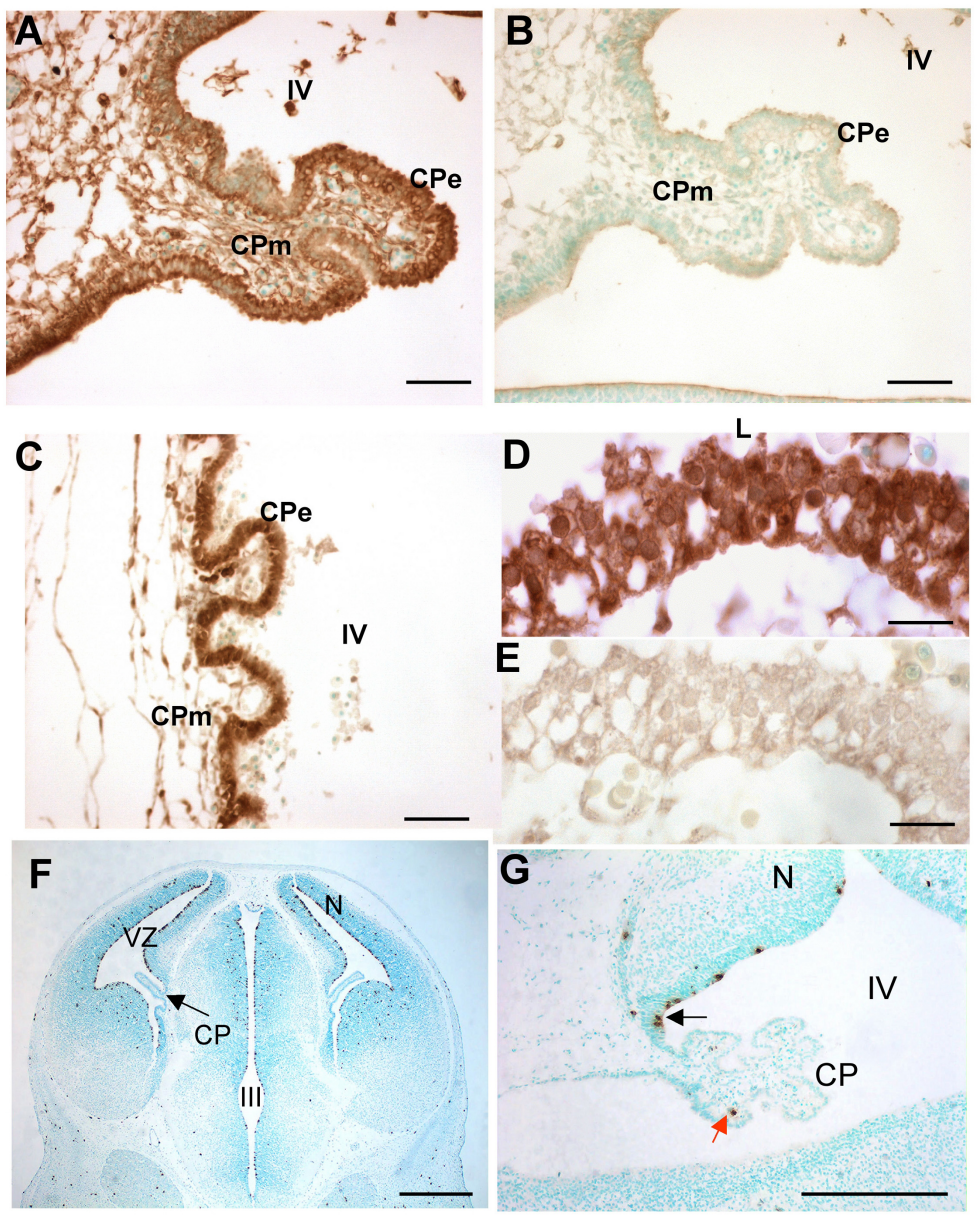
**Expression of Fgf2 and p-H3 in the mouse and human CPe**. (A) IV ventricle (IV) choroid plexus epithelium (CPe) in a sagittal section of E12.5 mouse embryo brain; positive cytoplasmic staining for Fgf2 protein is observed in the CPe and less strongly in the CP mesenchyme (CPm). (B) Negative control for (A). (C) Human IV ventricle CPe at 8 weeks of gestation: strong Fgf2 labelling is present in the CPe. (D) Human lateral ventricle CPe at 8 weeks of gestation; Fgf2 staining is mainly cytoplasmic. (E) Negative control for (D). (F) P-H3 staining of coronal section of E12.5 mouse embryo whole brain, including the choroid plexuses of the lateral ventricles. Neuroepithelial cells of the ventricular zone are frequently labelled with p-H3 antibody whereas p-H3 labelling in the CPs is rare. (G) Black arrow indicates p-H3 labelled cells in the IV ventricle CP stalk; red arrow indicates a p-H3 labelled cell in the CPm. Scale bars are 50 μm in A-B; 15 μm in C-D; 500 μm in F and 200 μm in G. CP = choroid plexus, CPe = choroid plexus epithelium, CPm = choroid plexus mesenchyme, L = lateral ventricle, N = neuroepithelium, VZ = ventricular zone, III = third ventricle, IV = fourth ventricle.

Previously, expression of Fgf2 in the CPe has not been studied at early developmental stages, nor across species. Therefore, this study investigated whether Fgf2 is present in the early embryonic mouse and human CP by immunohistochemistry. In the E12.5 mouse IVth ventricle CP, Fgf2 protein was strongly expressed in the epithelium and more weakly in the mesenchyme (Fig. 1A–B), and its expression was maintained at E16.5 (not shown). High levels of Fgf2 protein were also detected in both IVth and lateral ventricle CPe of human embryos at 8 weeks of gestation (Fig. 1C–E). To establish whether Fgf2 is expressed in dividing CP cells, we analysed E12.5 mouse IV and lateral ventricle CPe using the mitotic marker, p-H3 (phosphorylated histone 3). Very rarely p-H3 staining was observed in IVth and lateral ventricle CPe, whereas cells of the ventricular zone were frequently labelled (Fig. 1F–G). Some p-H3 positive cells were observed in the CP stalk of the IV ventricle (Fig. 1G). The noticeable difference in the expression patterns of Fgf2 and p-H3 suggests that the main role of Fgf2 during CPe development is not the control of CP cell proliferation.

### Effect of FGF2 on CPe proliferation and vesicle diameter

**Figure 2:**
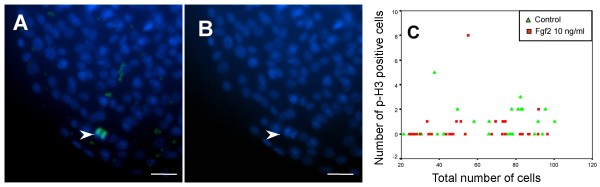
**Effect of Fgf2 on cell proliferation in CPe vesicles**. (A) Day 4 vesicles were stained for p-H3 and nuclei counterstained with Hoechst dye; the arrowhead points to a p-H3 stained cell. (B) Same field of view with Hoechst stain only. Scale bars: 25 μm. (C) Plot of the number of p-H3 labelled cells in day 4 vesicles either untreated or treated with 10 ng/ml Fgf2 against vesicle total cell number. Data are means ± SEM.

**Figure 3:**
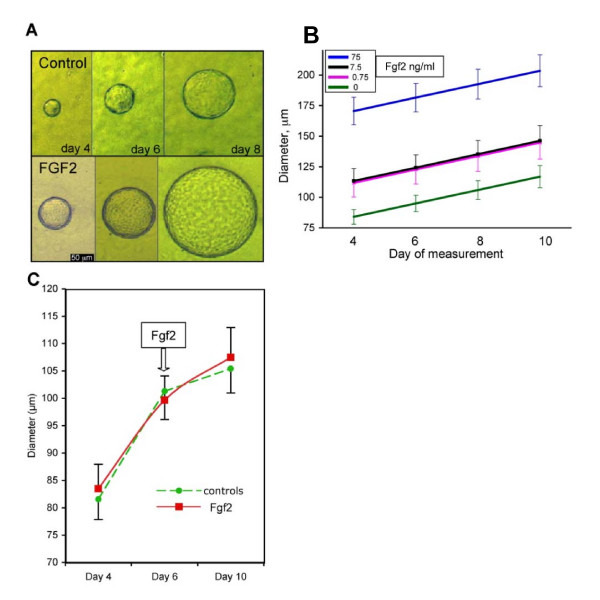
**Effect of Fgf2 on CPe vesicle size and growth**. Choroid plexus epithelial cells were cultured in Matrigel either in the presence or absence of Fgf2. (A) Micrographs of two vesicles, one control (upper panel) and one Fgf2-treated (7.5 ng/ml) taken at different times in culture (day 4, day 6 and day 8). (B) Graph showing the mean diameters of untreated vesicles (controls, n = 232) and vesicles treated with 0.75 (n = 63), 7.5 (n = 96) or 75 (n = 54) ng/ml Fgf2 measured on days 4, 6, 8 and 10. At all Fgf2 concentrations the vesicle diameter is significantly higher than in controls (p < 0.001). (C) Graph showing the average vesicle diameters of control vesicles (n = 96) and vesicles treated with 10 ng/ml Fgf2 from day 6 in culture (n = 110). The results were not significant. Data are means ± SEM.

As the CPe expresses Fgf2 and FgfRs, and at least FgfR2 is detected in CPe vesicles ([26] and unpublished observation), we further investigated the effect of Fgf2 on cell proliferation in primary CPe cells grown as monolayers and in CPe vesicles. We first studied the effect of different doses of Fgf2 on the proliferation of TRCSFB-2 cells (data not shown). Fgf2 significantly increased anti BrdU incorporation at 1 and 10 ng/ml compared to controls (*p *< 0.001). However, with increasing dose of Fgf2 the stimulation of cell proliferation declined and at the highest Fgf2 concentration tested (100 ng/ml) no difference in TRCSFB-2 proliferation was observed compared to untreated controls. Similarly, 10 ng/ml Fgf2 significantly increased cell proliferation in primary embryonic CPe cells grown as monolayers (*p *< 0.0001) (data not shown). Unlike in CPe monolayers, very few cells in control vesicles were mitotic (Fig. 2A–B), and the effect of 10 ng/ml Fgf2 on p-H3 expression was not significant either in 1 day cultures (not shown) or in 4 day vesicle cultures (Fig. 2C). Analysis of time lapse photography over 2 to 4 days, either at the time of plating or once vesicles had formed, did not provide any evidence of significant cell proliferation in Matrigel either in the presence or absence of Fgf2. Nonetheless, larger vesicles were observed in Fgf2-treated cultures than in controls (Fig. 3A). To further investigate Fgf2 effects on vesicle size, CPe cells plated in Matrigel were treated with 0.75, 7.5 or 75 ng/ml Fgf2 from the time of plating, and their diameter monitored over 8 days (Fig. 3A–B). By day 4 all Fgf2-treated vesicles were significantly larger than controls (Student's t-test *p *< 0.001 for every dose), and this effect was already significant at 0.75 ng/ml, the lowest concentration used (Fig. 3B).

Altogether these results demonstrate that while Fgf2 increases vesicle diameter, this is not due to increased proliferative activity of CPe cells. They also show that the behaviour of CPe cells in response to Fgf2 depends on the culture environment, given the difference in proliferative effect in monolayer and 3-dimensional (3D) cultures.

### Effect of Fgf2 on CPe vesicle secretion

Analysis of vesicle growth rate showed that the average rate of increase in diameter assessed between 4 and 10 days in culture was the same for control and Fgf2-treated CPe (Fig. 3B). This suggests that Fgf2 does not increase fluid secretion into the lumen, as it has been shown that vesicle growth rate is mainly due to secretion [30]. In addition, when Fgf2 was added to the cultures at 6 days (Fig. 3C), no significant difference in cell diameter between treated and untreated vesicles was observed at 10 days in culture.

**Figure 4:**
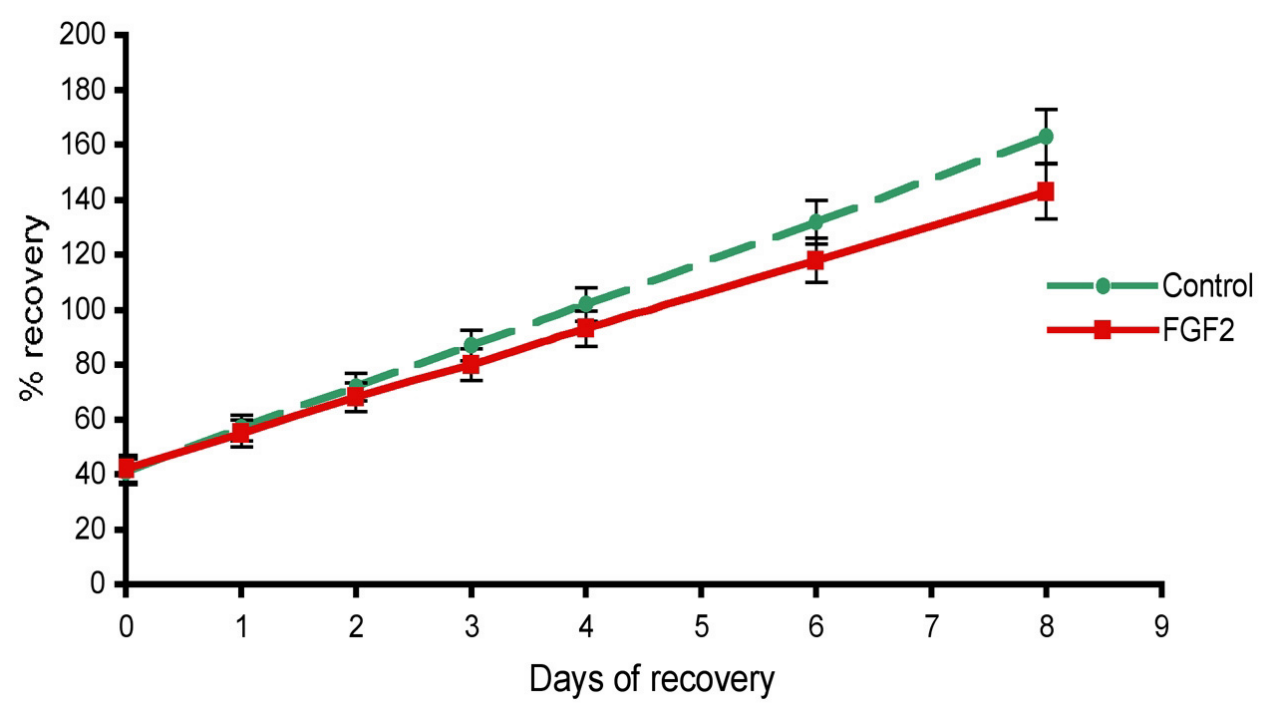
**Effect of Fgf2 on recovery of collapsed vesicles**. Graph showing the recovery of collapsed CPe vesicles in response to 10 ng/ml Fgf2 expressed as percentage of their size prior to collapse treatment with acetazolamide and ouabain (n = 110 per group). The extent of recovery between control and Fgf2-treated vesicles was not significantly different. Data are means ± SEM.

To further confirm that Fgf2 does not principally affect fluid secretion, we studied its effects on vesicle recovery after inducing them to collapse with secretion inhibitors. Vesicles were cultured for eight days, either with or without 10 ng/ml Fgf2, and then treated for two days with 5 mM ouabain and 100 μM acetazolamide. The effect of Fgf2 on the recovery in size of collapsed vesicles was then assessed by measuring their area daily over eight days (Fig 4). Since the original vesicles were of variable size, the recovery of each vesicle was expressed as percentage of its area before addition of ouabain and acetazolamide (% recovery). The recovery rates of the vesicles were compared between the two groups using a multilevel model (which accounted for the within vesicle correlation of repeat measurements). The average percentage increase in control vesicle size at each day was 1.19 times higher (95% confidence interval: 1.17, 1.20) than the previous day (p < 0.05). Fgf2-treated vesicles recovered slightly less rapidly than non-treated vesicles, but the difference between control and Fgf2 treatment groups was not significant. These results suggest that fluid secretion rate is not altered by Fgf2 treatment, and that the observed increase in vesicle size must be induced through different mechanisms at early stages of vesicle formation.

### Effect of Fgf2 on CPe vesicle formation

**Figure 5:**
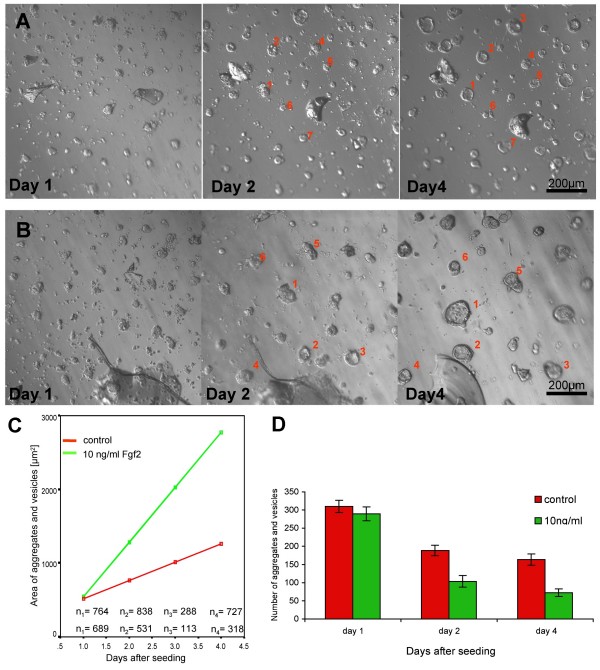
**Fgf2 effect on early CPe vesicle formation**. (A-B) Early formation of vesicles in the presence of 0 ng/ml (A) or 10 ng/ml (B) Fgf2 monitored in the same field of view at one, two and four days after plating in Matrigel. Note that on day 1, just before adding Fgf2 to the cells, CPe aggregates in (A) and (B) are similar in size. On day 2, Fgf2-treated CPe cells have formed bigger aggregates that are fewer in number compared to non-treated cells. On day 4, vesicles in the Fgf2-treated culture are larger than in control culture, but fewer in number. (C) Graph showing the average size of aggregates and vesicles in cultures treated without (control) or with 10 ng/ml Fgf2 at different times after seeding according to a multiple linear regression analysis. The fitted lines show that Fgf2-treated aggregates and vesicles become bigger in size over time. The average size difference between control and Fgf2 treated cultures is 462.5 μm^2 ^(SEM: 87.25 μm^2^), and Fgf2-treated aggregates increase every day on average by 492.03 μm^2 ^(compared to control aggregates. (D) Bar chart showing the mean number of aggregates and vesicles in cultures treated without or with 10 ng/ml Fgf2. At day 2 and day 4 after seeding, the number of aggregates and vesicles is significantly lower in Fgf2-treated cultures compared to controls (p < 0.05), whereas before the addition of Fgf2 on day 1, the difference is not significant. Data are means ± SEM.

Having established that Fgf2 treatment does not affect vesicle size by increasing proliferation or secretion and that its major effect on vesicle size occurs prior to day 4, we investigated the effects of Fgf2 on CPe behaviour at early stages of vesicle formation (Fig 5). To this purpose we treated CPe with 10 ng/ml Fgf2, starting the treatment one day after plating on to Matrigel. This allowed us to check that the cultures were at a similar density before addition of the growth factor, and to control for possible variability between experiments carried out from different preparations. The aggregates in Fgf2-treated cultures appeared already to be larger than controls after one day of treatment (2 days in culture), fusing with each other much more frequently (Fig 5A–B). After three to four days the aggregates became hollow and formed vesicles that were larger than those in control cultures. As shown by time-lapse photography, both treated and untreated vesicles were highly pulsatile (Additional files 1 and 2).

In order to quantify the effect of 10 ng/ml Fgf2, we measured the mid-plane area of CPe aggregates and vesicles in four separate cultures (Fig 5C). Regression analysis revealed that for every day of growth, Fgf2-treated aggregates and vesicles increased in size by an additional 492.03 μm^2 ^(SEM: 35.72) per day compared to control cultures, and that this effect was highly significant (p < 0.01). We quantified the number of aggregates forming in control and treated cultures, and found that their number was significantly lower in Fgf2-treated cultures than in controls both at one day (two days after plating) and 3 days (4 days after plating) treatment (Fig 5D), as expected given the greater number of cells recruited into each vesicle.

No obvious change in cell motility was observed when cells were treated with 10 ng/ml Fgf2 at the time of plating. Cell migration was further assessed by measuring the average speed of individual cells in control and 100 ng/ml Fgf2-treated cultures, to assess whether a change in motility might be more obvious at this higher dose that induces formation of larger vesicles. The speed of cell migration was computed by taking the total distance a cell migrated before incorporation into an aggregate over the amount of time taken to do so. In control cells the average speed was 20.29 ± 4.84 μm/h, and in Fgf2-treated cells the average speed was 20.48 ± 1.45 μm/h. The difference in cell motility was not significant. This suggests that changes in cell-cell adhesion, rather than cell motility, are likely to be responsible for the formation of bigger aggregates and consequently larger vesicles in Fgf2-treated cultures.

### Fgf2 regulation of genes associated with CPe function

**Figure 6:**
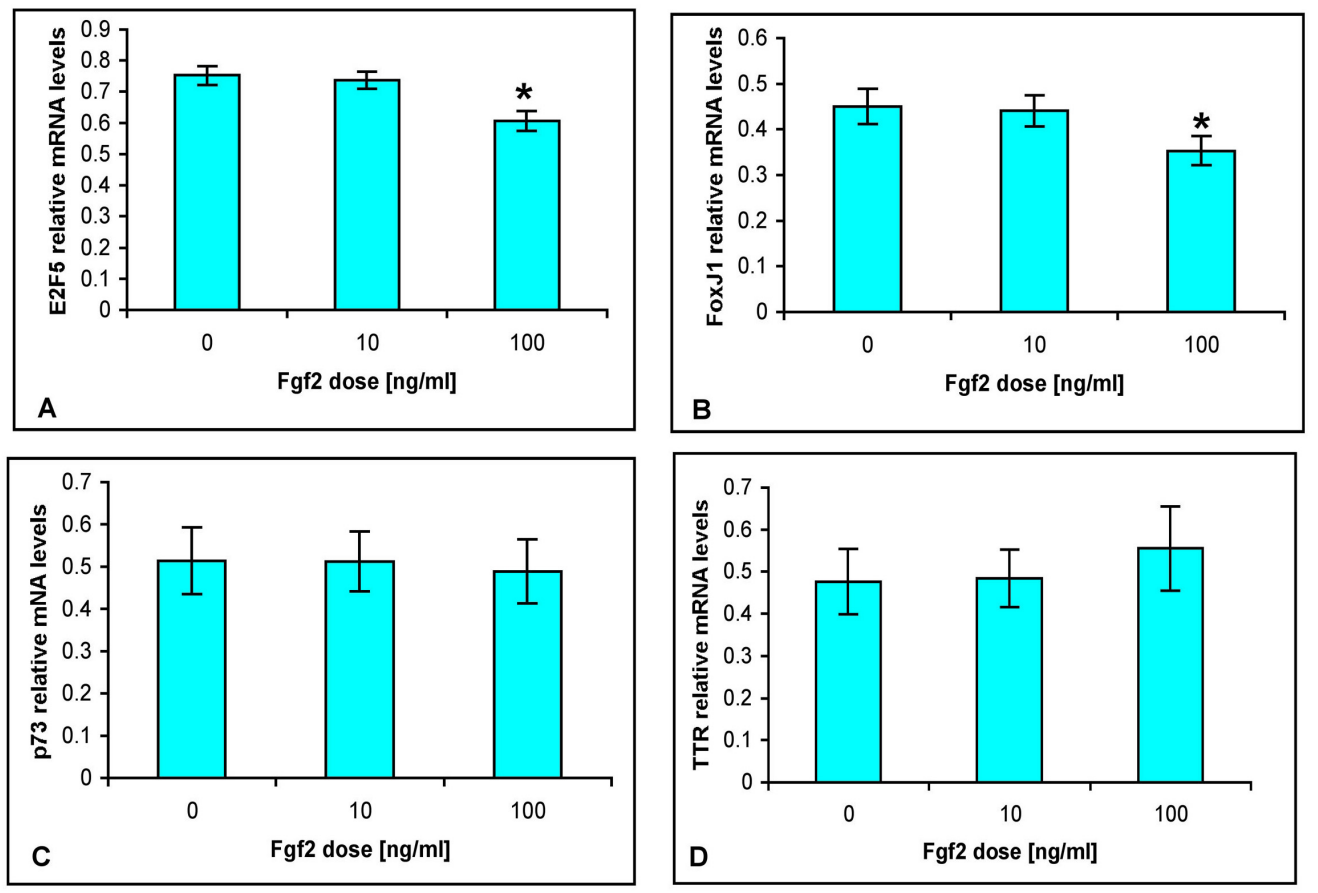
**Effect of Fgf2 on the expression of genes associated with CPe development and function in TRCSFB-2 cells**. The bar charts show the average level of mRNA expression for A: *E2f5 *(n ≥ 9), B: *FoxJ1 *(n ≥ 12), C: *p73 *(n ≥ 7) and D: *TTR *(n ≥ 11) in response to increasing Fgf2 concentrations (0 ng/ml, 10 ng/ml, 100 ng/ml). Expression of each gene was assessed by RT-PCR and normalised against expression of the housekeeping gene, *GAPDH*. Both *E2f5 *and *FoxJ1 *transcripts are significantly reduced by treatment with 100 ng/ml (p < 0.05). Data are means ± SEM.

In order to gain further insight into the possible function of Fgf2 we investigated its effects on the expression of E2f5, FoxJ1, p73 and transthyretin (TTR), a protein synthesized and secreted by the CPe. As the TRCSFB-2 cells appeared to behave in a similar fashion to primary mouse CPe monolayers when treated with Fgf2 and a large number of these cells can be easily grown, the effect of different concentrations of Fgf2 (1 ng/ml, not shown, 10 ng/ml and 100 ng/ml) on gene expression was assessed using semi-quantitative RT-PCR in TRCSFB-2 cells (Fig 6). A consistent reduction (p < 0.05) in *E2f5 *and *FoxJ1 *transcripts was induced by Fgf2 at the highest concentration tested, 100 ng/ml, (Fig 6A–B), but *TTR *and *p73 *expression was not significantly affected at any Fgf2 concentration (Fig 6C–D). These results suggest that Fgf2 may play a role in modulating CPe function.

## Discussion

### Fgf2 expression is an early event in CPe development

It was shown here that Fgf2 is expressed not only in mature CPe as previously reported [27,28], but also at early embryonic stages, and that such expression is conserved between mouse and human. As FgfRs are also expressed in the developing choroid plexus in both species [26,34], it is likely that Fgf2 can behave in an autocrine fashion *in vivo*, consistent with its ability to respond to Fgf2 *in vitro *demonstrated in this study.

Analysis of Fgf2 expression, cell division during development, and the effect of Fgf2 on CP cells cultured in Matrigel suggest that this growth factor does not play a key role in CPe growth. Whilst the concentrations of the growth factor used here (mainly 10 ng/ml) and in many *in vitro *studies may seem high in comparison to that in CSF *in vivo *[35], it is the concentration of Fgf2 at the cell surface which is critical [12,36-38]. As discussed above, autocrine and paracrine Fgf signalling is likely to occur in the CPe, and Fgf2 availability is regulated by the extracellular matrix. Although in our 3D culture system the bioavailability of Fgf2 may be modified as a result of interactions with the extracellular matrix, a clear effect on vesicle size was observed at all the concentrations tested.

Whereas CP cells in Matrigel are not induced to proliferate in response to Fgf2, CP cells grown as monolayers, either on laminin or collagen, significantly increase their proliferative activity in the presence of low/medium Fgf2 concentrations. This different proliferative response may be largely due to the differences in culture architecture in the two models. CPe morphology is different in these two culture conditions, with cells being initially rounded and then tightly packed to form a simple polarized epithelium in Matrigel, and well-spread and flat in monolayers. An environment-dependent proliferative effect of Fgf2 on CPe cells is also consistent with previous work showing that these cells behave differently when plated on different matrices [39]. These authors tested growth factors present in trace amounts in Matrigel, Tgfβ1, Tgfβ2 and Pdgf, for their ability to increase penetration of CPe into matrices, and suggested that Tgfβ2, but not Tgfβ1 and Pdgf, can increase migration depending on matrix composition. A role of the environment in Fgf2-induced proliferative response is also consistent with *in vivo *studies reporting increased proliferation in Fgf2-treated CPe in pathological conditions, such as in a Parkinson's disease model [23], but no obvious size changes in the CPe of normal animals that developed Fgf2-induced hydrocephalus [23].

### Fgf2 increases CPe cell aggregation rather than secretion

It has been previously shown that growth of CPe vesicles depends on fluid secretion within the lumen [30]. Analysis of vesicle growth rate has shown that Fgf2-induced increase in vesicle size is not due to increased secretion into the lumen. The slopes of the growth curves of vesicles exposed to different Fgf2 concentrations are very similar, and no increase in the rate of recovery of vesicles collapsed using secretion inhibitors is induced by Fgf2 treatment. In addition, if Fgf2 treatment is started at 6 days, once the vesicles have already formed, no increase in vesicle size is observed in treated cultures 4 days later. Transmission electron microscopy analysis of Fgf2-treated vesicles (not shown) is also consistent with lack of significant Fgf2 effects on secretion, as no obvious reduction in electron density, which is thought to decrease in CPe with very high secretory activity, is observed in these vesicles. Our results *in vitro *are consistent with work suggesting that the occurrence of hydrocephalus observed in response to Fgf2 treatment *in vivo *is not caused by a direct effect of Fgf2 on CPe secretion, but could be due, at least in part, to a decrease in CSF absorption caused by impaired function of the arachnoid villi, the main structure involved in this process [23].

Fgf2-induced vesicle growth is due to increased CPe cell recruitment into the aggregates that form vesicles, as indicated by a reduced number of aggregates and increase in aggregate size in the presence of Fgf2. The mechanisms underlying increased cell recruitment leading to formation of larger aggregates have yet to be fully elucidated, but the present results suggest an increase in cell-cell adhesion rather than in cell motility. Fgf2-treated aggregates appear more compact than controls. Time-lapse microscopy analysis has not indicated any obvious increase in cell motility in the presence of Fgf2 and, as discussed earlier, proliferation does not appear to contribute to increased aggregate size. E-cadherin could be a candidate mediator of the effect of Fgf in the CPe, as it is expressed in adult CP and Fgf treatment can upregulate E-cadherin expression in pancreatic cancer cell lines [40-42]. A thorough adhesion molecule profile of the CPe is required to identify putative Fgf targets leading to increased cell aggregation.

The fact that Fgf2 is expressed early during choroid plexus development and appears to affect CPe cell-cell interaction, rather than cell proliferation, is consistent with a role of Fgf2 in maintaining the integrity of this epithelium as recently suggested for endothelial cells of the blood brain barrier [36].

### Fgf2 modulates expression of transcription factors associated with CPe homeostasis

The effect of Fgf2 on *E2f5 *and *Foxj1 *is of particular interest, given the putative roles of these transcription factors in the CPe. Knockouts of *E2f5*, a member of a family (*E2f1–E2f6*) of transcriptional regulators and *FoxJ1*, a member of the forkhead-box (Fox)/winged helix gene family, have been associated with defective choroid plexus function resulting in hydrocephalus [4-6]. Foxj1 is associated with ciliogenesis and maturation of CPe [4,9,43], and Fgf2-induced down-regulation of *Foxj1 *has been reported in other cell types [12]. In addition, defects in cilia formation have been recently reported in Fgf10 null mutants, supporting the view that Fgf signalling may play a role in ciliogenesis [44]. *E2f5 *is a general cell cycle regulator, but in the CPe seems associated with CPe function rather than proliferation [5,7]. Since E2f5 intracellular localization changes with CPe maturation, and the FgfRs are differentially regulated during its development [7,26], it is conceivable that Fgf signalling may play a role in modulating translocation of this protein from the nucleus to the cytoplasm in the developing CPe.

The dose-dependent effects of Fgf2 on proliferation (induced at low/medium concentrations), and gene expression (induced at high concentrations) in the CPe cell line are akin to those observed in other cell types, such as fibroblasts and cranial neural crest cells, where at lower concentrations Fgf2 stimulates proliferation, whereas at higher concentrations it appears to support survival and differentiation [37,45].

## Conclusion

Altogether, this study suggests that whereas Fgf2 does not have a significant direct effect on CSF secretion, it is an endogenous regulator of the Fgf signalling pathway in the developing choroid plexus, given its conserved expression in rodents and humans. Furthermore, Fgf2 is likely to play a role in maintaining CPe integrity and function, as suggested by the increase in CP cell aggregation and modulation of gene expression observed *in vitro*. In order to better understand the role of Fgf2 on cell-cell interaction it will be important to identify which cell-cell adhesion molecules might be modulated by Fgf2 in the CPe either at the mRNA or protein level and other Fgf ligands that may be crucial for Fgf signalling in the CPe.

## Competing interests

The authors declare that they have no competing interests.

## Authors' contributions

SG and AS carried out the experimental work, contributed to its design, interpretation of the results and to the first draft of the manuscript. AW advised on the statistical analysis and supervised SG and AS on this aspect of the work. TT developed the cell line used in this study and gave final approval to the manuscript. PF conceived, designed and funded the study, carried out data analysis and interpretation of the results, and finalized the manuscript. All authors have read and approved the final version of the manuscript.

## Supplementary Material

Additional file 1 Time-lapse of control CPe vesicle. Two days time-lapse of control CPe vesicle cultures which had been grown for 8 days in Matrigel. Click here for file

Additional file 2 Time-lapse of Fgf2-treated CPe vesicle. CPe vesicles grown in the presence of 10 ng/ml Fgf2. Pictures of the same field of view were taken at 1 h intervals. Both treated and untreated vesicles are highly pulsatile. Click here for file
